# Undifferentiated carcinoma with osteoclast-like giant cells of the pancreas: a narrative review

**DOI:** 10.3389/fonc.2024.1409197

**Published:** 2024-06-19

**Authors:** Haoxiang Wu

**Affiliations:** Fudan University Shanghai Cancer Center, Shanghai, China

**Keywords:** pancreatic cancer, variants, undifferentiated carcinoma, osteoclast-like giant cells, prognosis

## Abstract

Undifferentiated carcinoma with osteoclast-like giant cells of the pancreas (UCOGCP) is a rare pancreatic tumor that accounts for less than 1% of all pancreatic malignancies. The characteristic pathological manifestation of UCOGCP is the presence of osteoclast-like giant cells (OGCs) distributed among pleomorphic undifferentiated tumor cells. UCOGCP can occur either alone or in association with other types of pancreatic tumors. At present, there is no unified consensus or guideline for the diagnosis and treatment of UCOGCP, and most of the literature are individual case reports. With the accumulation in the number of clinical cases and the development of precision medicine technology, the understanding of UCOGCP is also deepening. Researchers have begun to recognize that UCOGCP is a pancreatic tumor with distinctive clinical and molecular characteristics. In this review, we focus on the latest research status and future exploration directions in the diagnosis, treatment, and prognosis of UCOGCP.

## Introduction

**Figure 1:**
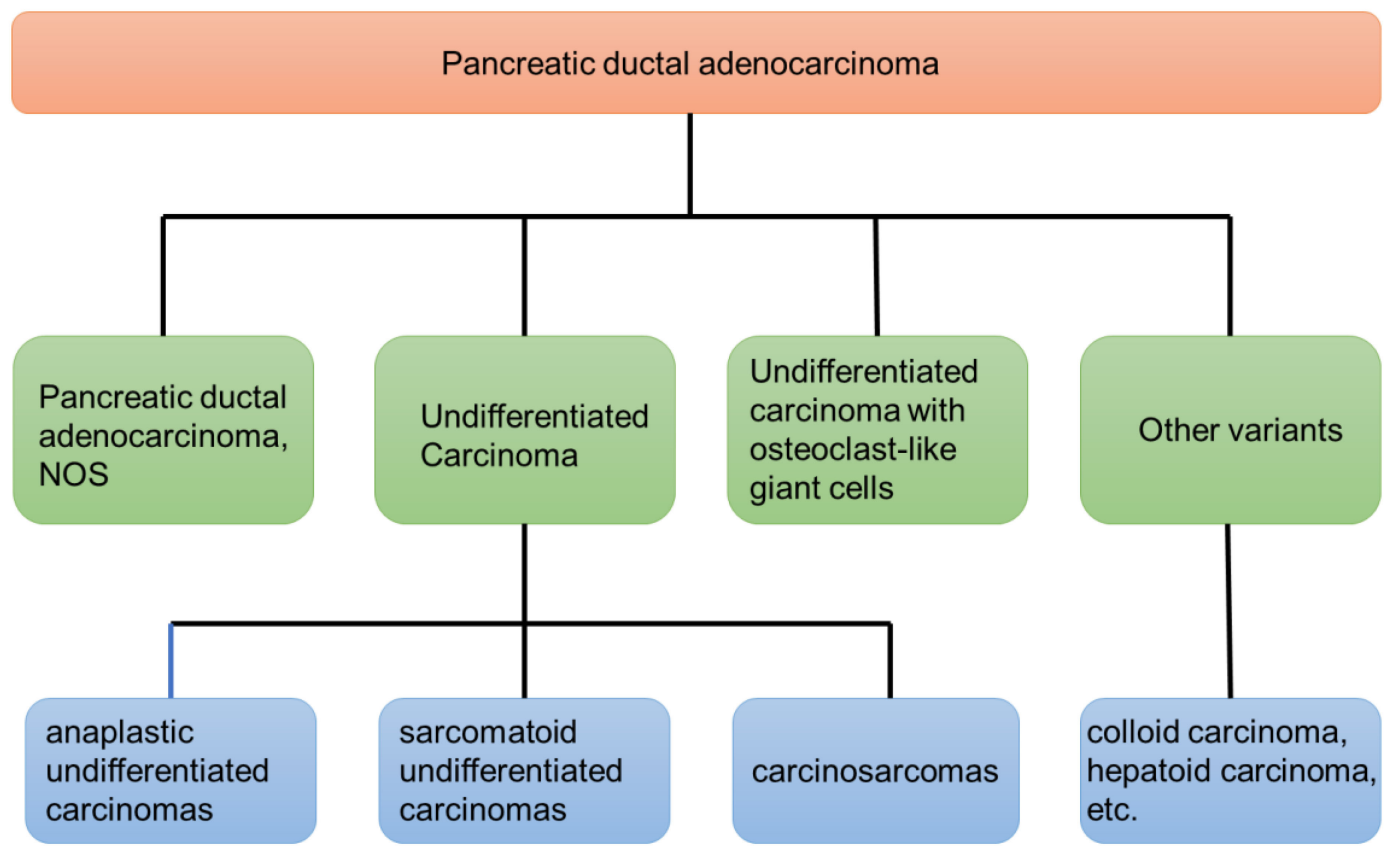
The latest WHO’s classification of pancreatic ductal adenocarcinoma (2019).

Pancreatic ductal adenocarcinoma (PDAC) is a malignancy associated with an overall 5-year survival rate of approximately 10% (1). There are several variants of PDAC based on histopathological characteristics. Among these variants, UCOGCP is a rare entity with unique clinical and pathological features. Tumors containing osteoclast-like giant cells occurring outside the bone are relatively rare. They are gradually being discovered in various parts of the body such as the pancreas, kidney, breast, and thyroid gland, with the pancreas being the most common site of occurrence. The primary pathologic characteristic is the presence of osteoclast-like giant cells within the tumor tissue which are morphologically similar to giant cell tumors of bone. UCOGCP is a rare non-endocrine malignant tumor of the pancreas and is easily confused with other types of pancreatic malignant tumors. The first description of this tumor was proposed by Sommers and Meissner in 1954 (2). Juan Rosai later notified that this tumor simulated giant cell tumors of bone in 1968 (3). According to the 4th edition of the WHO classification, undifferentiated carcinoma of pancreas (UCP) is divided into four types: anaplastic type, sarcomatoid type, carcinosarcoma, and undifferentiated carcinoma with osteoclast-like giant cell (4). UCP accounts for 2–7% of pancreatic malignant tumors (5, 6), while UCOGCP accounts for less than 1% of pancreatic malignant tumors (6–8). The 2019 WHO classification classified UCOGCP and UCP as independent subtypes of pancreatic ductal adenocarcinoma (9). More details about the 2019 WHO classification can be seen in **Figure 1**. Refined classification of pancreatic adenocarcinoma is important because the clinical course and outcome of each subtype differ significantly. PDAC and pancreatic neuroendocrine tumor (PNET) both have generally accepted management guidelines, but UCP does not. There are relatively few existing research articles on UCOGCP, most of which are retrospective case reports. So far, only slightly more than 100 global reports have been retrieved. The individual cases of UCOGCP published over the past five years are summarized in **Table 1**. Little is currently known about UCOGCP. The histogenesis and biology of this type of tumor has not yet been fully unraveled. This article aims to introduce the latest status and future prospects of diagnosis and treatment of UCOGCP.

**Table 1:** Individual case reports on UCOGCP over the past five years.

Author	Age	Sex	CA199	CEA	Size(cm)	Location	Meta	Survival	Status	Ref
Cai	54	Male	NA	NA	20.4	tail	0	84	Alive	(10)
Yang	31	Male	NA	NA	3	uncinate process	0	12	Dead	(6)
Gao	78	Male	NA	NA	0.7	uncinate process	1	10	Dead	(11)
Smith	69	Male	NA	NA	13	periampullary	0	NA	NA	(12)
Olayinka	NA	Male	Normal	NA	7.2	body	0	3	Alive	(13)
Cavalcanti	65	Male	93.8	NA	3	head	0	5	Alive	(14)
Yamamoto	71	Male	Normal	Normal	4	head	0	20	Alive	(15)
Besaw	62	Female	NA	NA	4.9	tail	1	32	Alive	(16)
Jiang	62	Male	NA	NA	10	tail	1	NA	NA	(17)
Sozutek	52	Female	0.8	4.4	5	uncinate process	0	20	Alive	(18)
Talakić	47	Male	NA	NA	5.8	body	0	NA	NA	(19)
Kitazono	58	Female	24.3	1.1	2	body	0	60	Alive	(20)
Ashfaq	79	Male	534	NA	2	head	0	NA	Loss	(21)
Gupta	56	Male	73	NA	7	body and proximal tail	1	2	Dead	(22)
Tomihara	68	Male	7.6	2.4	9.9	tail	0	NA	NA	(23)
Igarashi	63	Male	147	3.7	7.5	head and body	0	6	Alive	(24)
Jain	62	Male	42	NA	1	body	0	NA	NA	(25)
Swaid	76	Male	NA	NA	8.2	body and tail	1	6	Dead	(26)
Chan	54	Male	138	2.4	11	tail	0	11	Dead	(27)
Ran	78	Male	1968	NA	4.5	body and tail	0	6	Dead	(28)

Meta, metastasis at diagnosis; NA, not available/applicable.

## Diagnosis

### Clinical characteristics

UCOGCP has a wide age range of onset and is more commonly observed in middle-aged and elderly people (29). Most studies believe that there is no significant gender difference in the incidence of UCOGCP (5, 30), although some studies showed that it is slightly more common in women (14, 31, 32). In terms of clinical symptoms and signs, UCOGCP is similar to PDAC. Non-specific clinical presentations include abdominal pain, abdominal mass, jaundice, weight loss, etc. Among them, abdominal pain or abdominal discomfort is the most common (29). Some patients do not even have any clinical symptoms. Specific clinical symptoms are related to the location of the tumor and its size. In terms of tumor serological characteristics, tumor markers such as carcinoembryonic antigen (CEA) and CA19–9 are either not significantly elevated or fall within the normal reference range (33). Elevated CA19–9 levels in some UCOGCP patients may be associated with the presence of ductal adenocarcinoma components within the tumor. Some reports indicate an increase in neuron-specific enolase (NSE) levels in certain UCOGCP patients (30).

### Imaging characteristics

In clinical practice, the diagnosis of UCOGCP mainly relies on histopathological examination of preoperative biopsies or postoperative specimen and there are no specific imaging findings. Preoperative abdominal ultrasound, contrast-enhanced CT, MRI and other imaging examinations can clearly delineate the size and location of the tumor, which complement each other and contribute to the clear diagnosis of malignant tumors. According to existing reports, UCOGCP mostly presents as cystic-based or mixed cystic-solid mass with delayed enhancement (13, 31, 34), and some cases are purely solid or purely cystic. The masses are frequently accompanied by necrosis and hemorrhage (35). The borders are mostly clear, and the surrounding invasion is not obvious. UCOGCP can occur in any part of the pancreas. Most tumors are located in the head of the pancreas (8, 34).Tumors located in the pancreatic head and neck can result in dilation of the pancreatic duct. It is noteworthy that tumor volume of UCOGCP is usually larger (8), with 80% larger than 5cm and 50% larger than 10cm (33). Analysis of the reasons may be related to the following aspects: (1) Tumors grow quickly; (2) Many tumors are located in the body and tail of the pancreas, with relatively low malignancy and late onset of clinical symptoms; (3) Tumor volume is often accompanied by bleeding which often leads to an increase in tumor size. Endoscopic ultrasound (EUS) is a great tool for the diagnosis of UCOGCP. In EUS examination, PDAC is uniformly hypoechoic, while UCOGCP has uneven echo (36). EUS-guided fine needle aspiration (FNA) and fine needle biopsy (FNB) are able to acquire tumor tissue and further help perform immunohistochemical analysis, which is the only method to diagnose UCOGCP before surgery (8). However, preoperative FNA will increase the incidence of postoperative complications and requires careful selection. Meanwhile EUS-guided FNA can sometimes give the wrong diagnosis. In Muraki’s study, of the 15 UCOGCP patients who underwent preoperative lesion biopsy, only 4 accurate diagnoses were achieved (32). PET-CT can provide useful information about the metabolic status of tumors and the presence of distant metastasis (37). The newer endoscopic ultrasound-guided fine-needle biopsy (EUS-FNB) combined with next-generation sequencing technology also facilitates related diagnosis (38, 39).

### Pathological characteristics

UCOGCP is an aggressive non-endocrine tumor of pancreas with no glandular differentiation. Histopathological and immunohistochemical classification remains the gold standard for the diagnosis of UCOGCP. Microscopically, its cellular components are mainly composed of neoplastic mononuclear cells and numerous distinctive multinucleated giant cells. These giant cells resemble the giant cells of the bone which is why they are also called osteoclast-like giant cells. The multinucleated OGCs are considered non-neoplastic because they do not share the molecular aberrations of the neoplastic pleiomorphic mononuclear cells. It is often accompanied by massive hemorrhage and necrosis, which may affect the results of pathological diagnosis. OGCs usually have densely eosinophilic cytoplasm and multiple relatively uniform and bland nuclei clustering in the central aspect of the cell (32). Immunohistochemically, OGCs express CD68, does not express cytokeratin AE1/AE3, and has a very low Ki-67 proliferation index (14, 40). Pleomorphic tumor cells are positive for Vimentin, usually express markers of epithelial differentiation (cytokeratin, EMA), may have mutated p53 immuno-profile, and have a high Ki-67 proliferation index (41, 42). Most cases exhibit prominent intraductal/intracystic growth (32). The histogenesis of UCOGCP is still controversial. From the immunohistochemistry results, monocytes showed characteristics of epithelial origin, and OGCs showed characteristics of mesenchymal origin. Most current studies believe that it is of epithelial origin (7, 8). Most UCOGCP are accompanied by KRAS gene mutations, which also supports the origin of ductal epithelial cells (43, 44). There are also reports of lesions originating during epithelial-to-mesenchymal transition (EMT) (45). Through machine learning of gene expression patterns between UCOGCP samples and PDAC samples, it was found that OGCs originate from stem cell-like mesenchymal epithelial cells (SMEC) (46). UCOGCP can be accompanied by other epithelial tumors, PDAC is the most common, and 75% of cases can be accompanied by PDAC at the same time (8, 32). It can also be accompanied by mucinous cystic neoplasm (MCN), intraductal papillary mucinous neoplasm (IPMN) or malignant stromal tumors (15, 31, 47, 48). **Table 2** shows literature reports on UCOGCP with mixed components. If no other distinct epithelial tumor components can be found, it’s called pure UCOGCP. Through analysis of the immunohistochemical expression of three well-known EMT markers, EMT activation was found more frequent in anaplastic carcinomas than in UCOGCP (58).

**Table 2:** Reports of UCOGCP associated with other types of tumors.

Author	Year	Cases	Details	Ref
Nai	2005	1	1 with MCN	(49)
Sedivy	2005	1	1 with MCC	(50)
Pan	2007	1	1 with MCN	(51)
Burkadze	2009	1	1 with MCN	(52)
Wada	2011	1	1 with MCN	(53)
Chiarelli	2015	1	1 with MCN	(47)
Muraki	2016	38	29 with PDAC, 4 with MCN, 4 with IPMN	(32)
Reid	2017	12	8 with PDAC, 2 with MCN, 1 with IPMN	(8)
Luchini	2017	22	10 with PDAC, 2 with MCN, 1 with IPMN	(54)
Luchini	2018	27	16 with PDAC	(42)
Hanayneh	2019	1	1 with MCN	(55)
Charifa	2019	1	1 with IPMN	(56)
Farhat	2019	1	1 with jejunal GIST	(57)
Gao	2020	1	1 with NET	(11)
Yamamoto	2021	1	1 with IPMN	(15)
Mattiolo	2021	16	7 with PDAC	(58)
Smith	2021	1	1 with PDAC	(12)
Olayinka	2021	1	1 with PDAC and focal signet ring features	(13)
Sozutek	2022	1	1 with PDAC	(18)
Gao	2022	13	3 with PDAC	(30)
Zhao	2023	4	2 with PDAC, 1 with ductal mucinous adenocarcinoma	(48)
Chung	2023	1	1 with IPMN	(59)
Hrudka	2024	13	3 with PDAC, 2 with IPMN	(60)

MCN, mucinous cystic neoplasm; MCC, mucinous cystadenocarcinoma; PDAC, pancreatic ductal adenocarcinoma; IPMN, intraductal papillary mucinous neoplasm; GIST, gastrointestinal stromal tumor; NET, neuroendocrine tumor.

### Genetic characteristics

Modern gene sequencing technologies such as next-generation sequencing (NGS) have been widely used. In addition to long-term follow-up, investigation of the genomic alterations in confirmed and surviving patients can also help guide the selection of treatment options and prognostic analysis. To better understand UCOGCP, many genetic features have been identified. The most frequently mentioned gene mutation of UCOGCP in literature reports is the *KRAS* gene (59, 61). Most *KRAS* mutations were found in codon 12, including the G12V, G12D and G12R mutations. *KRAS* is one of the most common oncogenes in human cancer. *KRAS* activation is an early event in the pathogenesis of pancreatic cancer. *KRAS* mutations are present in approximately ~85% of PDAC patients (62). Whole-exome sequencing (WES) of eight cases showed that UCOGCP has surprisingly similar molecular characteristics compared with conventional PDAC (54). In addition to the common *KRAS* oncogenic mutations, other typical mutated genes including *TP53*, *CDKN2A* and *SMAD4* were found in UCOGCP, which further confirm that UCOGCP is a unique variant of PDAC. Mutations in *SERPINA3*, *GLI3*, *MAGEB4*, *MEGF8* and *TTN* have also been detected. Some of them may act as pathogenic or likely pathogenic mutations. Zhao et al. detected 6 potentially clinically significant genes through NGS: *AR, FBXW7, CCNE1, BTK, KRAS, TP53* (48). Somatic *BRCA2* alterations were also detected by WES in one case (6). Recently, Hrudka et al. performed molecular genetic analysis of 13 UCOGCP cases with varying survival time (60). This is the largest NGS examined UCOGCP cohort to date. The results showed that the mutant spectrum of UCOGCP is very similar to that of PDAC. Unfortunately, UCOGCP-specific genomic signatures remain mainly unknown and no somatic genetic aberrations were found that were clearly related to patient prognosis or PD-L1 status. Gene characteristics of UCOGCP in literatures are summarized in **Table 3**. MicroRNAs (miRNAs) play a variety of important regulatory roles in both healthy and cancer cells. Popov et al. evaluated the expression of seven previously clinically significant miRNAs in UCOGCP and poorly differentiated (grade 3, G3) PDACs (65). Except for miR-155, there were no significant differences in the expression of other miRNAs in the two tumors.

**Table 3:** Reports on UCOGCP gene characteristics.

Author	Year	Cases	Results	Ref
Imai	1999	1	*KRAS*	(63)
Sedivy	2005	1	*KRAS*	(50)
Koorstra	2008	1	*CDKN2A*	(64)
Luchini	2017	8	*KRAS*(G12V)*, CDKN2A, TP53, SMAD4, SERPINA3, MAGEB4、GLI3、MEGF8、TTN*	(54)
Charifa	2019	1	*KRAS* (G12D)*, SMAD4* (C127F), and a *TP53* splice site mutation	(56)
Yang	2020	1	*KRAS* (G12D)*, BRCA2*	(6)
Smith	2021	1	*KRAS* (G12D)*, TP53*(C176F)*, IDH2*(G144A)	(12)
Yamamoto	2021	1	*KRAS* (G12V) and *GNAS* (R201C)	(15)
Cavalcanti	2021	1	*KRAS* (G12V)*, BRAF*	(14)
Jain	2022	1	*KRAS, TP53*	(25)
Zhao	2023	1	*AR、FBXW7、CCNE1、BTK、KRAS、TP53*	(48)
Chung	2023	1	*KRAS* (G12D)	(59)
Hrudka	2024	13	*KRAS, TP53, CDKN2A, SMAD4, CIC, GNAS, APC, ATM, NF1, FBXW7, ATR*, and *FGFR3*	(60)

## Treatments

### Surgery

Regarding UCOGCP, because its incidence is very low, most literatures are case reports, there are few randomized controlled studies of large cohorts, and there are no relevant guidelines for reference. Consequently, the clinical course of this disease remains poorly understood. Overall, radical surgical resection with negative margins (R0 resection) is the optimal treatment when surgical indications are met. The selection of surgical method and the scope of lymph node dissection can refer to PDAC, which mainly depends on the location of the tumor, regional lymph node involvement and intraoperative exploration. If nearby organs such as the stomach, jejunum, colon, and kidneys are invaded, they may need to be resected together. Because this type of tumor has large lesions and rich blood supply, careful preoperative imaging evaluation can help improve the complete resection rate and safety of surgery.

### Chemotherapy

Pancreatic cancer is the most aggressive solid tumor in humans with a very poor prognosis. The treatment of pancreatic cancer is based on tumor histology. Because UCOGCP is so rare, the optimal treatment approach for this kind of tumor has not yet been determined. Several randomized clinical trials have confirmed that patients with PDAC at all stages need to receive chemotherapy. Since UCOGCP is a variant of pancreatic ductal adenocarcinoma, the common chemotherapy regimen for PDAC such as FOLFIRINOX(combination of oxaliplatin, irinotecan, fluorouracil and leucovorin) and gemcitabine can be often seen in case reports of UCOGCP. On the other hand, it has been reported in the literature that the comprehensive treatment strategy for UCOGCP can refer to undifferentiated pancreatic cancer. And a retrospective multicenter cohort study suggests that for patients with UCP, nab-paclitaxel-based combination chemotherapy may be more advantageous and significantly improved OS compared with non-paclitaxel-containing regimens (6.94 months vs. 3.75 months, respectively; P = 0.041) (66). For unresectable locally advanced or borderline resectable UCOGCP, there are reports in the literature that FOLFIRINOX regimen and modified FOLFIRINOX can be used for downstaging before surgery (18, 24). Neoadjuvant chemotherapy (NACT) can help reduce tumor size and alleviate local progression, thereby creating opportunities for R0 resection of the tumor without the need for resection of major blood vessels or adjacent organs. And it can also select patients with good tumor biological characteristics, who will benefit from curative resection surgery.

### Immunotherapy

For PDAC, immunotherapy regimens have always had poor efficacy, while there are relatively few reports on the application of immunotherapy in UCOGCP. UCOGCP seems to exhibit high expression of universal programmed death ligand-1 (PD-L1), which may be used as an effective prognostic marker (41, 42, 67). PD-L1 expression was more frequent in UC compared with conventional PDAC (63% vs 15%, P < 0.01) (67). For UCOGCP, PD-L1 was expressed on tumor cells in 17 of 27 cases (63%) and was more often in patients associated with PDAC (42). Moreover, multivariate analysis confirmed that PD-L1 expression was associated with poor prognosis (HR = 3.397; 95% CI, 1.023–18.375; P = 0.034). The reason behind this may be that PD-L1 suppresses anti-tumor immunity and allows tumor cells to evade the cytotoxic activity of host T lymphocytes.

Another study including 13 UCOGC patients demonstrated similar conclusions (41). UCOGC expressed PD-L1 significantly more frequently and had greater numbers of CD3+ and CD8+ tumor-infiltrating lymphocytes (TILs) compared with PDAC. Among the 3 PD-L1 negative UCOGCP cases, none of them reached the death end point. The median survival time of PD-L1-positive patients was 5.7 months. However, due to the small cohort size, the comparison was not statistically significant. Immunotherapy may exert anti-tumor effects on UCOGCP with distant metastasis. Obayashi et al. (68) reported a UCOGCP patient with pancreatic body and tail tumors and lung metastases who achieved clinical complete remission of lung metastases after monotherapy with pembrolizumab monoclonal antibody. The patient achieved disease-free survival after subsequent resection of the pancreatic body and tail tumors. For a patient with unresectable UCOGCP with lung metastases, whose tumor mutation burden (TMB) was high as measured by NGS, he started to receive third-line pembrolizumab monotherapy after palliative radiotherapy. After 32 months of treatment, the primary tumor and metastases achieved sustained remission (16). So far, the effectiveness of immunotherapy in patients with UCOGCP still needs to be verified in large cohorts and more clinical trials.

## Prognosis

### Prognostic data with varying outcomes

Reports on UCOGCP are scarce and the terminology and classification of UCOGCP are not uniformly standardized. As a result, the prognostic data is fairly limited and vary greatly. Large survival differences may be related to tumor tissue heterogeneity and different tumor stages at diagnosis. Overall, UCOGCP is a highly aggressive tumor. UCOGCP lacks typical clinical symptoms and imaging features, and has poor tissue differentiation and strong invasiveness. UCOGCP is often diagnosed in the advanced stages with large tumor size, and many patients cannot get complete surgical resection. There is also a lack of effective therapeutic drugs. Even if it can be resected, early recurrences are also common. So the overall prognosis is poor. The opinion given in the WHO 2019 Digestive System Tumors Volume is that patients may survive for 1 year or more (9). The survival time of UCOGCP reported in most existing literature also shows similar or better results (30, 41, 69). Based on the data analysis of UCP patients in the National Cancer Database (NCDB), the median survival time of UCOGCP patients was 24.8 months and the estimate 3-year and 5-year survival rates were 42.54% and 39.01%, respectively (70). The difference in survival is also related to the stage of the tumor. By integrating previous literature reports, the median overall survival time of UCOGCP patients who underwent surgical resection was found to be 48 months (29). For unresectable UCOGCP patients with locally advanced tumors or distant metastasis, the prognosis is significantly worse and median survival time is less than 6 months (71, 72). **Table 4** lists more detailed prognostic data of UCOGCP based on reported case series.

**Table 4:** Reports on UCOGCP survival data based on case series.

Author	cases	media os(months)	os(surgery)	os(without surgery)	3-year os(%)	5-year os(%)	Ref
Clark	11					50	(5)
Muraki^a^			92			59.1	(32)
Luchini	16	20					(54)
Reid	15	8					(8)
Xu	47	13	33	5			(73)
Hrudka	13	8.4					(41)
Shiihara	113	26	48			46.8%^b^	(29)
Imaoka^c^	13	5.36					(72)
Kharkhach		11					(74)
Ueberroth	15	11					(69)
Christopher	119	24.8			42.54	39.01	(70)
Gao	13	13					(30)

OS, overall survival.

a. This study included 38 resected UCOGCP and 725 resected PDAC.

b. This 5-year survival rate was calculated using the data of patients who underwent surgical resection.

c. This study included 13 unresectable UCOGCP.

### Pure UCOGCP seems to have a better prognosis

Pure UCP is highly malignant and has a poor prognosis. It is worth noting that UCOGCP is slightly less malignant than conventional PDAC and undifferentiated carcinoma without OGCs. In Strobel’s study, median survival for patients with anaplastic pancreatic cancer was 5.7 months, compared with 15.7 months for control patients with pancreatic ductal adenocarcinoma (75). The survival times of the three UCOGCP patients were even significantly longer, 33, 49, and 161 months respectively. The tumor resection rate of UCOGCP was also unexpectedly found to be higher than other types of UCP (29). In a recent large database review of UCP, patients with OGCs (UCOGCP) had longer median OS (aHR 0.52: 95% CI 0.41–0.67) compared with patients without OGCs (70). A proportion of UCOGCP patients in previous publications are still alive many years after diagnosis. A patient with locally advanced UCOGCP survived for even 10 years after undergoing surgery and adjuvant therapy (33). In one literature report, the patient’s survival time was even 15 years (76). Despite its rapid growth and large tumor size, the biological behavior of UCOGCP is different from that of common PDAC (77). Although UCOGCP grows rapidly, recent reports suggest that perineural invasion and lymph node metastasis are rare, and its prognosis is relatively better than that of PDAC and pleomorphic giant cell tumor (32, 78). According to Muraki’s study, the five-year survival rate of resected UCOGCP is 59.1%, while the five-year survival rate of resected PDAC is 15.7% (32). However, some research results showed that the prognosis of UCOGCP patients may be worse than that of PDAC patients, and there is a lack of effective large sample data to further clarify this issue (30).

However, some literature demonstrated different results. One retrospective study evaluated 55 patients with unresectable UCP. Final survival analysis showed that there is no significant difference in OS between the UC with OGCs group and the UC without OGCs group (72). Apart from tumor stages at diagnosis, the possible reasons behind may be related to the pathological components and the different roles of OGCs. More detailed pathological differences may have an impact on the prognosis of UCOGCP patients. As mentioned before, UCOGCP can be categorized into 2 distinct pathological subtypes: pure UCOGCP, and UCOGCP associated with other epithelial tumors. Patients with UCOGCP alone seem to survive longer than patients with UCOGCP accompanied by PDAC (30, 32, 54, 79). A meta-analysis using previous reported data showed that major prognostic factors of UCOGCP include age, sex, tumor size, lymph node metastasis, and concomitant PDAC component (79). However, concomitant MCN component did not affect prognosis.

## Conclusions and future perspectives

UCOGCP is an extremely rare pancreatic malignant tumor with no specific clinical manifestations, imaging features and blood tumor markers. The diagnosis mainly relies on postoperative pathology examination. Currently, the underlying mechanism of UCOGCP’s distinctive morphological features remains incompletely understood. Additionally, prognostic information also needs to be further explored. In the future, a lot of further research work can be carried out to prolong the survival time of UCOGCP patients. In terms of clinical research, more cases and long term follow-up data can be collected to obtain more detailed information. At the same time, the level of early detection and diagnosis should be improved, and the treatment regimen should be unified and standardized. In terms of basic research, more latest technological means such as artificial intelligence and multi-omics can be used to explore the underlying pathogenesis and provide more precise and effective treatment strategies.

## Author contributions

HW: Writing – original draft, Writing – review & editing.
